# Intracellular Pathogen *Leishmania donovani* Activates Hypoxia Inducible Factor-1 by Dual Mechanism for Survival Advantage within Macrophage

**DOI:** 10.1371/journal.pone.0038489

**Published:** 2012-06-12

**Authors:** Amit Kumar Singh, Chaitali Mukhopadhyay, Sudipta Biswas, Vandana Kumari Singh, Chinmay K. Mukhopadhyay

**Affiliations:** Special Centre for Molecular Medicine, Jawaharlal Nehru University, New Delhi, India; University of Oklahoma Health Sciences Center, United States of America

## Abstract

Recent evidence established a crucial role for mammalian oxygen sensing transcription factor hypoxia inducible factor-1 (HIF-1) in innate immunity against intracellular pathogens. In response to most of these pathogens host phagocytes increase transcription of HIF-1α, the regulatory component of HIF-1 to express various effector molecules against invaders. *Leishmania donovani* (LD), a protozoan parasite and the causative agent of fatal visceral leishmaniasis resides in macrophages within mammalian host. The mechanism of HIF-1 activation or its role in determining the fate of LD in infected macrophages is still not known. To determine that J774 macrophages were infected with LD and about four-fold increase in HIF-1 activity and HIF-1α expression were detected. A strong increase in HIF-1α expression and nuclear localization was also detected in LD-infected J774 cells, peritoneal macrophages and spleen derived macrophages of LD-infected BALB/c mice. A two-fold increase in HIF-1α mRNA was detected in LD-infected macrophages suggesting involvement of a transcriptional mechanism that was confirmed by promoter activity. We further revealed that LD also induced HIF-1α expression by depleting host cellular iron pool to affect prolyl hydroxylase activity resulting in to stabilization of HIF-1α. To determine the role of HIF-1 on intracellular LD, cells were transfected with HIF-1α siRNA to attenuate its expression and then infected with LD. Although, initial infection rate of LD in HIF-1α attenuated cells was not affected but intracellular growth of LD was significantly inhibited; while, over-expression of stabilized form of HIF-1α promoted intracellular growth of LD in host macrophage. Our results strongly suggest that LD activates HIF-1 by dual mechanism for its survival advantage within macrophage.

## Introduction

The oxygen sensing transcription factor hypoxia-inducible factor-1 (HIF-1) is a heterodimer of regulatory subunit HIF-1α and constitutive HIF-1β [1]. In oxygen deficiency or cellular iron depletion, expression of HIF-1α is regulated by a post-translational protein stability mechanism mediated by a family of prolyl hydroxylases (PHDs) [2]. HIF-1α subunit has a very short half-life (∼2 min) because it is targeted by an oxygen-dependent mechanism to the proteasome by the von Hippel-Lindau (VHL) E3 ubiquitin ligation [3]. The recognition of HIF-1α by VHL depends on hydroxylation of two proline residues (pro402 and pro564) by three HIF-1α prolyl hydroxylases (PHD1-3) but PHD2 was found as the primary isoform responsible for this hydroxylation mechanism [4]–[6]. In general, PHDs hydroxylate HIF-1α using oxygen and 2-oxoglutarate as substrates and iron and ascorbate as essential cofactors [6], [7]. Upon exposure to hypoxia or iron depletion PHD activity is affected resulting into stabilization of HIF-1α, which in turn translocates to the nucleus and forms a dimer with HIF-1β to activate HIF-1. Once activated, HIF-1 binds to the hypoxia response elements (HREs) of target genes implicated in metabolism, angiogenesis, apoptosis and cellular stress [8]. Recent evidences suggest that HIF-1 plays a novel and important role in infections and inflammatory diseases [9], [10]. HIF-1 activation was reported to be essential for bactericidal capacity of phagocytes by producing several immune effector molecules for host defense [11]. In fact, HIF-1 activation was reported as a general phenomenon in infections with human pathogens [12]. In response to pathogens, HIF-1 expression is upregulated through pathways involving key immune response regulator NFκB [10]. Lipopolysaccharide (LPS), the bacterial membrane component of gram negative bacteria activates HIF-1 in macrophages by NFκB dependent transcriptional mechanism [13], [14]. Interestingly, the basal expression of HIF-1α is also regulated by NF-κB [15] and this evolutionary conserved link between NF-κB and HIF-1 provides a strong innate immunity mechanism to phagocytes against invading pathogens [10], [15], [16].

**Figure 1:**
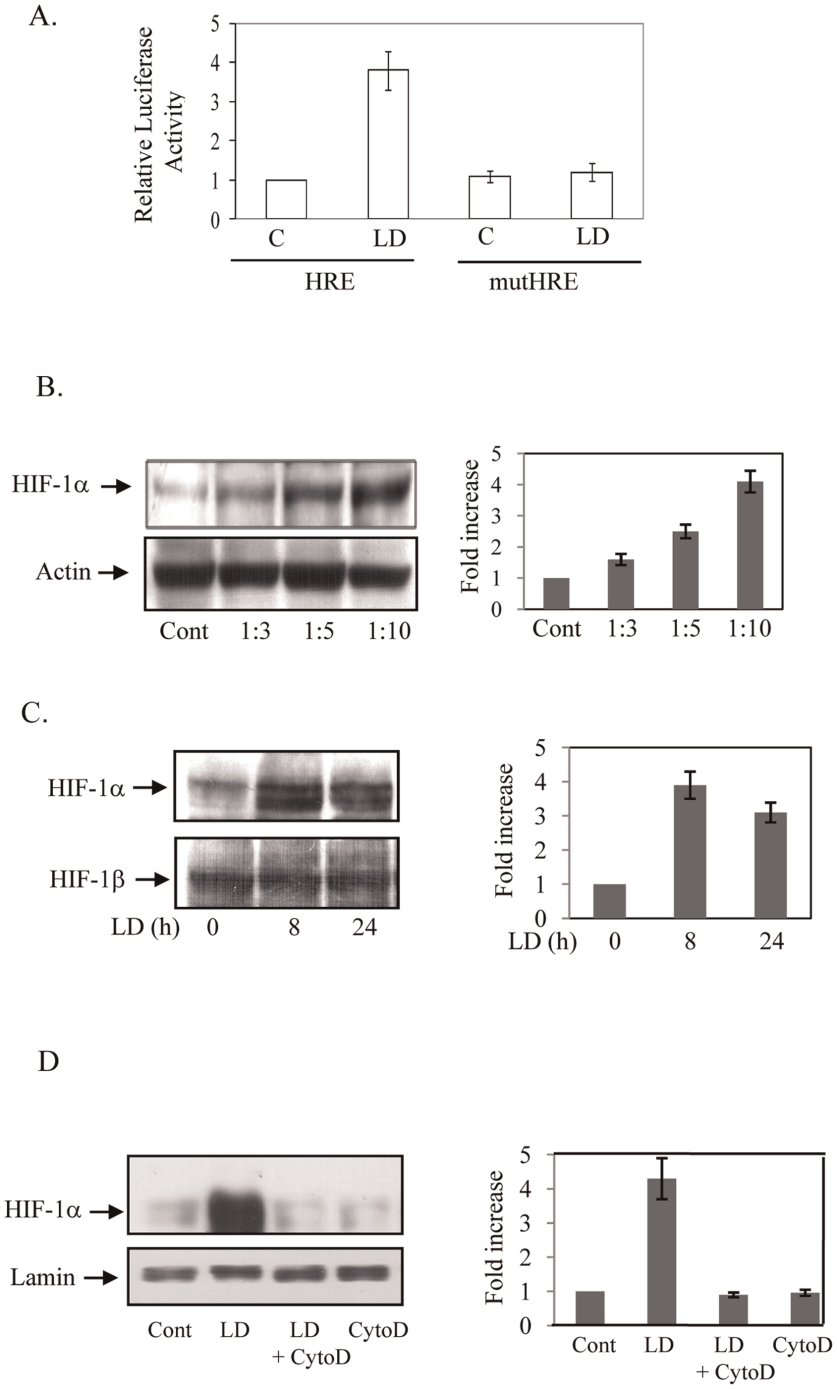
LD activates HIF-1 in macrophages in vitro. A. J774 cells were transfected either with wild type HRE or mutated HRE and β-galactosidase constructs. After 16 h of infection with LD (MOI-1∶10, macrophage: LD) luciferase activity in cell extracts was measured and normalized with β-galactosidase activity. Results are represented as SD of 3 independent experiments performed in triplicate. B. Western blot analyses for HIF-1α (left upper panel) and Actin (left lower panel) were performed in nuclear extracts isolated from J774 cells infected by LD with different ratios of multiplicity for 16 h. Right panel represents densitometric analysis from three independent experiments. C. Similar experiments were performed after 0, 8 and 24 h of infections with LD (MOI-1∶10) and Western blot analyses were performed for HIF-1α (left upper panel) and HIF-1β (left lower panel). Right panel represents densitometric analysis from three independent experiments. D. J774 cells was incubated with cytochalasin D (2 µM; CytoD) 60 min before LD infection and Western blot analysis was performed for HIF-1α (left upper panel) and lamin (left lower panel) in nuclear extracts isolated after 8 h. Right panel represents densitometric analysis from three independent experiments.

*Leishmania donovani* (LD), a digenetic protozoan parasite infects and resides within macrophages during its mammalian cycle of existence resulting into visceral leishmaniasis (VL). VL might be fatal if not treated properly and was reported to cause mortality in several parts of the developing world [17]. *Leishmania* infection is also detected as coinfection in HIV patients [17]. After successful entry into macrophages, promastigote form of the parasite survives and proliferates within the mature phagolysosome compartment as an amastigote, multiplies within and finally burst the host to infect neighboring macrophages [18]. During its stay within parasitophorous vacuoles (PV) of macrophages, parasite scavenges nutrients from the host cell, prevents host cell apoptosis, and alters host cell gene expression [19]. Therefore, leishmania has developed mechanisms to manipulate host cell processes that permit the parasite to grow within hostile environment within host macrophages. Recently, expression of HIF-1α is reported in *L. amazonensis* infected skin lesions by immunocytochemistry [20], [21] but its role in controlling leishmanial infection or its mechanism of activation remains to be resolved.

**Figure 2:**
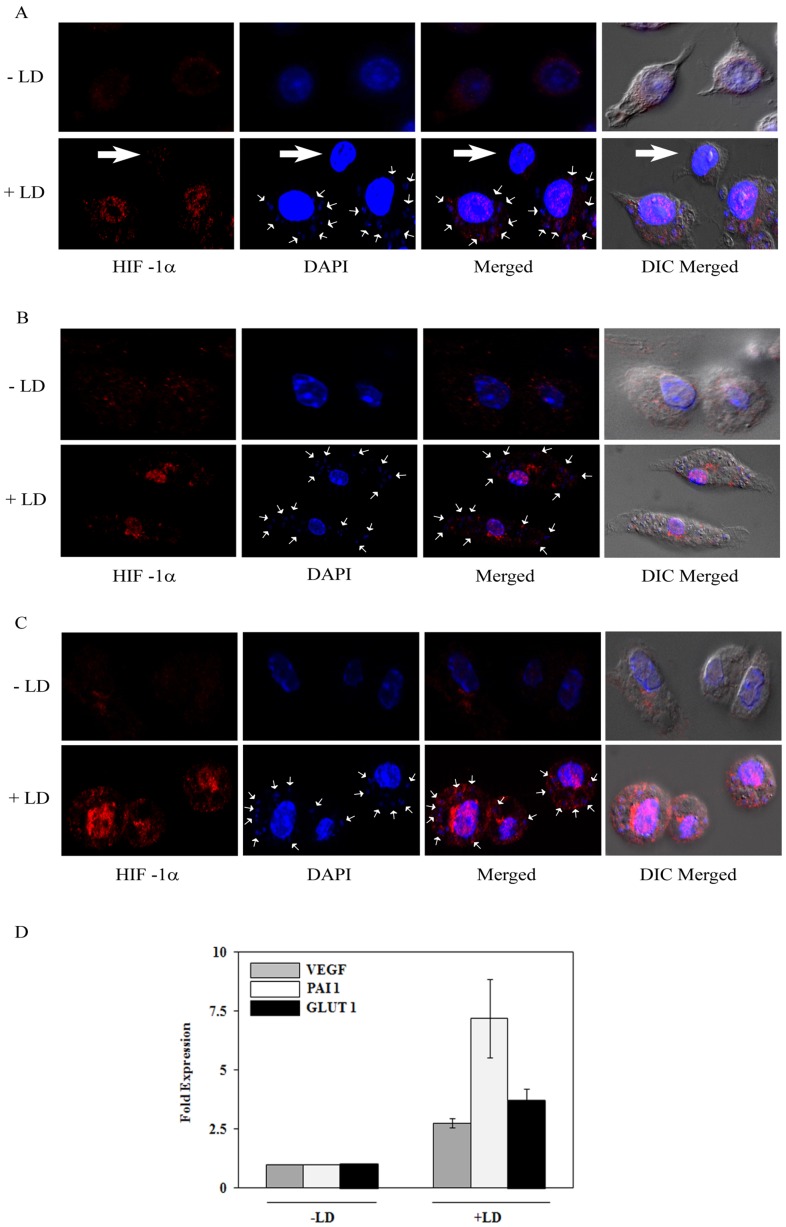
LD promotes HIF-1α nuclear localization in macrophages in vitro and in vivo. A. J774 cells were infected with LD (MOI-1∶10) (lower panel) or remain uninfected (upper panel). After 6 h of infection indirect immunofluorescence assay was performed using HIF-1α antibody. DAPI was used for nuclei staining of the host and intracellular LD (indicated by white arrows). In lower panel bigger and horizontal white arrow represents the J774 cell that was not infected with LD. Result is representative of one of six independent experiments. B. Peritoneal macrophages were isolated from BALB/c mice and infected with LD (MOI-1∶10). After 6 h of infection indirect immunofluorescence assay was performed using HIF-1α antibody. DAPI was used for nuclei staining of both the host and LD (indicated by white arrows). Result is representative of one of the four independent experiments. C. Splenic macrophages were isolated from uninfected (-LD) or LD infected BALB/c mice (+ LD) and HIF-1α was detected by indirect immunofluorescence assay using HIF-1α antibody. DAPI was used for detection of nuclei of both host and LD (indicated by white arrows). D. Real-time RT-PCR was performed from total RNA isolated from splenic macrophages isolated from LD-infected or uninfected mice using specific primers for HIF-1 target genes like VEGF, PAI-1, GLUT-1. β-actin was determined as a control. Results are representative of three independent experiments in each of which total RNA was isolated from macrophages derived from spleen of three mice.

The goal of this work is to determine the mechanism by which LD increases HIF-1α protein level and to find that whether HIF-1 is beneficial or detrimental to the invading parasite. We reveal the involvement of HIF-1α transcription and protein stability mechanisms in HIF-1 activation in LD infected macrophages. We also report that HIF-1 activation is advantageous for survival and growth of the parasite within host macrophage as knocking down of HIF-1α by specific siRNA affects intracellular survival and growth of the parasite. Other way, over-expression of stabilized form of HIF-1α promotes growth of intracellular LD suggesting a pivotal role of HIF-1 in benefiting intracellular pathogen LD.

**Figure 3:**
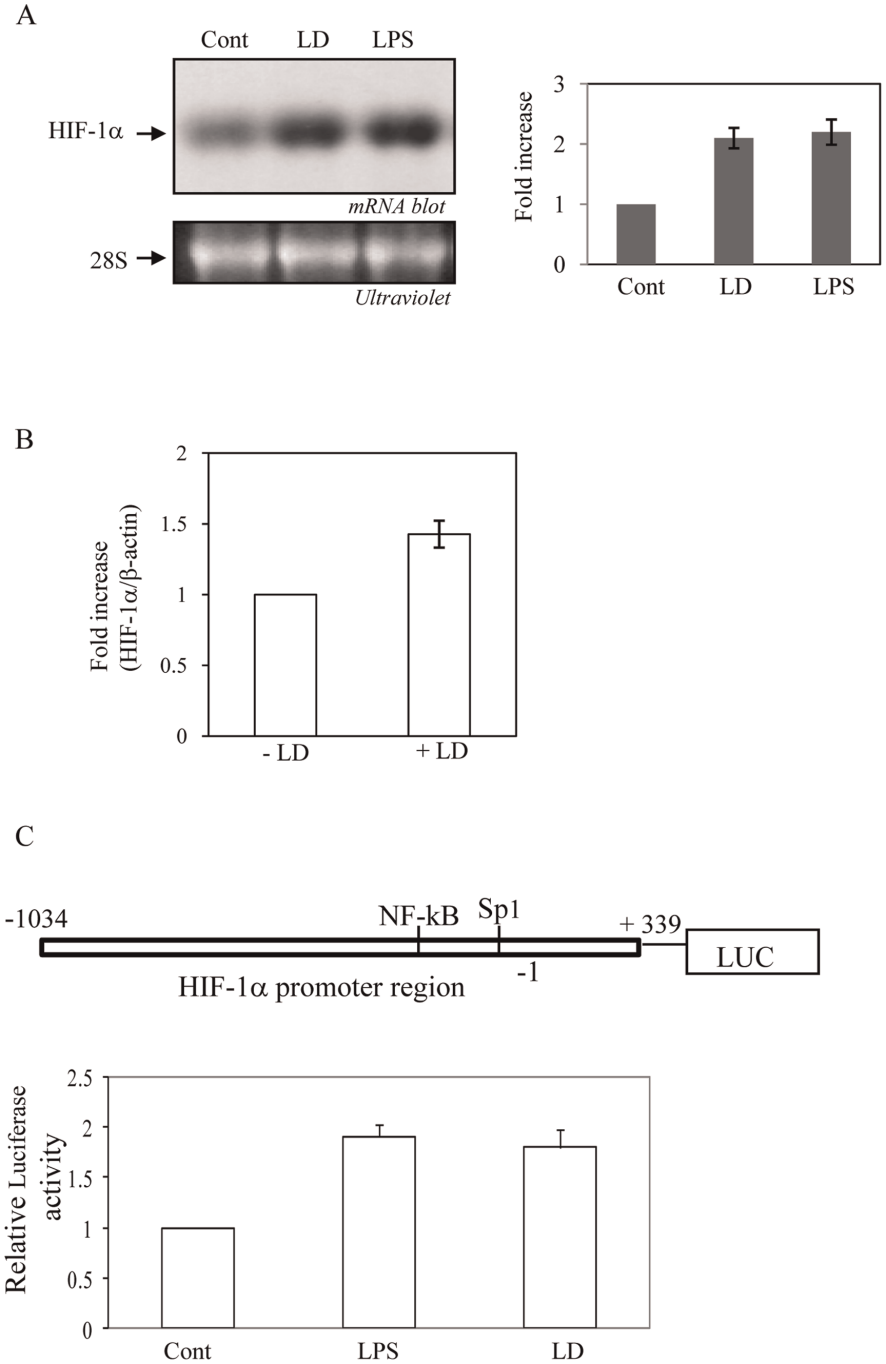
Transcriptional activation of HIF-1α by LD infection. A. Northern analysis of HIF-1α was performed (left upper panel) with total RNA isolated from LD infected (MOI-1∶10) and LPS (1 µg/ml) treated J774 cells (8 h). 28S rRNA detected using ultraviolet served as loading control (left lower panel). Right panel represents densitometric analysis from three independent experiments. B. Total RNA was isolated from spleen derived macrophages from uninfected and LD-infected mice and real time RT-PCR was performed using either mouse HIF-1α (upper panel) or mouse β-actin (lower panel) specific primers obtained from Applied Biosystems. Data is representative of one of the four different experiments (n = 4). C. A luciferase chimera with HIF-1α promoter and β-galactosidase with SV40 promoter constructs were transfected in J774 cells and either incubated with LPS (1 µg/ml) or infected with LD (MOI-1∶10). After 12 h luciferase and β-galactosidase activities were determined in cell lysates. Results were expressed as SD of three independent experiments performed in triplicates after normalizing luciferase activity with β-galactosidase activity.

## Results

### Activation of HIF-1 in Macrophages by LD Infection

To determine the influence of LD infection on HIF-1 activation, J774 macrophages were transfected with an active HRE driven luciferase construct (CpHRE) [22] and then infected with virulent LD (MOI-1∶10). About four fold increase in luciferase activity was detected by LD infection (Fig. 1A). However, transfection of a mutant HRE-luciferase construct (mut-CpHRE) [22] showed no change in luciferase activity (Fig. 1A) suggesting LD-infection resulted in HIF-1 activation in J774 macrophages. Western blot analysis was performed with nuclear extracts isolated from LD-infected J774 cells to confirm that HIF-1 activation was actually because of increased expression of HIF-1α. A steady increase up to about 4-fold in HIF-1α protein level was detected with increased multiplicity of infection of LD (Fig. 1B). A strong increase of HIF-1α was detected within 8 h of infection that remained increased even after 24 h of infection but expectedly no change in HIF-1β was detected (Fig. 1C). To determine whether internalization of LD was required for increased HIF-1α expression cytochalasin D was used to block parasite internalization by phagocytosis as described earlier [23]. Initially we confirmed the ability of cytochalasin D (2 µM) to block internalization of LD in J774 macrophages (data not shown). In a similar condition we detected a complete blocking of HIF-1α expression in J774 cells when incubated with LD (Fig. 1D). In a complimentary experiment conditioned medium of parasite was incubated with J774 macrophages to detect whether any soluble factor released by the parasite was responsible for HIF-1α expression in host. No change in HIF-1α expression was detected by conditioned medium of the parasite (data not shown). Together these results strongly suggest that internalization of LD is necessary for increased HIF-1α expression in host macrophages. To further detect the effect of LD-infection on nuclear localization of HIF-1α of the host macrophages, indirect immunofluorescence assay was performed in J774 and peritoneal macrophages isolated from BALB/c mice (Fig. 2A & 2B). Results showed a strong nuclear localized HIF-1α only in LD-infected macrophages. This was best depicted in Fig. 2A showing the cell not infected with LD (bigger- horizontal white arrow in lower panel) did not show nuclear localization of HIF-1α, while other two adjacent cells infected with LD (small white arrows) were detected with nuclear localized HIF-1α. This result further suggests that LD does not produce any soluble factor for HIF-1α expression as described earlier. A similar result was also obtained in LD-infected RAW 264.7 macrophages (data not shown). To further confirm that LD-infection promotes HIF-1 during *in vivo* infection, macrophages were isolated from LD infected BALB/c mice and prominent nuclear localized HIF-1α immunofluorescence was detected in LD-infected macrophages (Fig. 2C). Further, increased expressions of several HIF-1 target genes like VEGF, GLUT-1 and PAI-1 were detected in macrophages isolated from LD-infected BALB/c mice than uninfected mice by real-time RT-PCR (Fig. 2D). These experiments suggest LD activates HIF-1 in host macrophages both *in vivo* and *in vitro* conditions.

### LD Infection Increases HIF-1α mRNA and Protein Stability in Macrophages

Bacterial infection and LPS treatment increase HIF-1α expression by promoting transcription of HIF-1α; whereas, other inducers of HIF-1 like hypoxia, iron depletion or cobalt stabilize HIF-1α protein by decreasing prolyl hydroxylase activity [4], [5]. To determine whether any of these mechanisms was responsible for LD-induced activation of HIF-1, J774 cells were infected with LD and Northern blot analysis was performed. LPS was used as a positive control. About 2 fold increase in HIF-1α mRNA was detected by LD infection (Fig. 3A) indicating HIF-1α mRNA expression was at least partially responsible for LD-induced HIF-1 activation. An increase in HIF-1α mRNA was also observed by real-time RT-PCR in macrophages isolated from LD-infected mice (Fig. 3B) confirming this mechanism is also operative during *in vivo* infection. To confirm the involvement of transcriptional mechanism, we cloned HIF-1α promoter in pGL3-basic vector upstream of luciferase gene, transfected into cells and performed luciferase assay after LD infection or LPS treatment. Simultaneously, β-galactosidase under the control of SV40 promoter [24] was cotransfected to monitor transfection efficiency. About 2 fold increase in luciferase activity by LD infection confirmed the involvement of HIF-1α transcription for HIF-1 activation in host macrophage (Fig. 3C).

**Figure 4:**
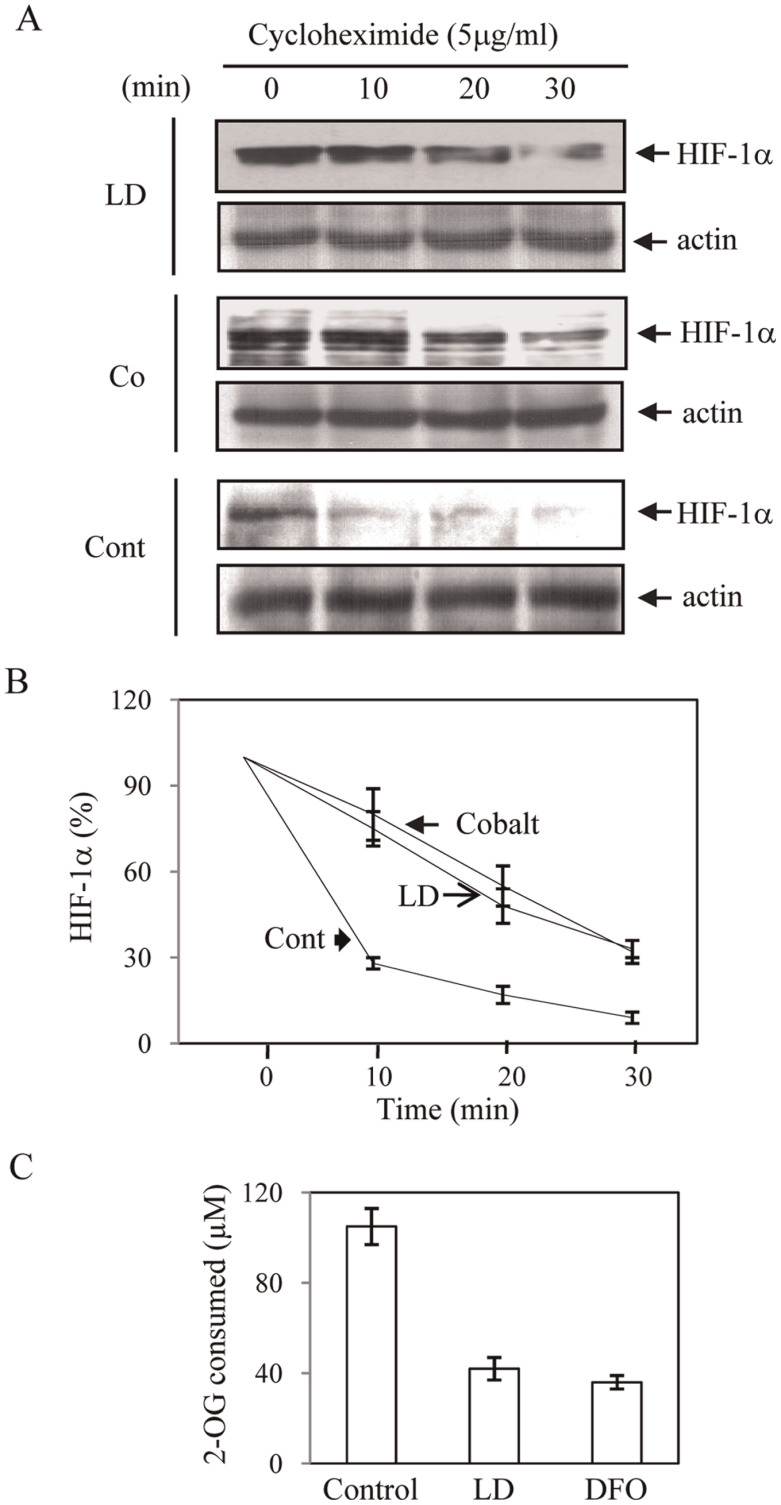
LD infection promotes HIF-1α stability. A. J774 cells were infected with LD or remained uninfected or treated with hypoxia mimetic cobalt chloride (100 µM) for 6 h and then cycloheximide (5 µg/ml) was added. Nuclear extracts were isolated after 0, 10, 20 and 30 min of cycloheximide addition and Western blot analyses for HIF-1α and Actin were performed. B. Relative stabilization of HIF-1α was determined by densitometric analysis of three independent experiments as described in ‘A’. C. Prolyl hydroxylase assay was performed as a measure of 2-OG consumption in cytoplasmic extracts after 8 h of LD infection and DFO (100 µM) treatment. Result is expressed as standard deviation of four different experiments.

**Figure 5:**
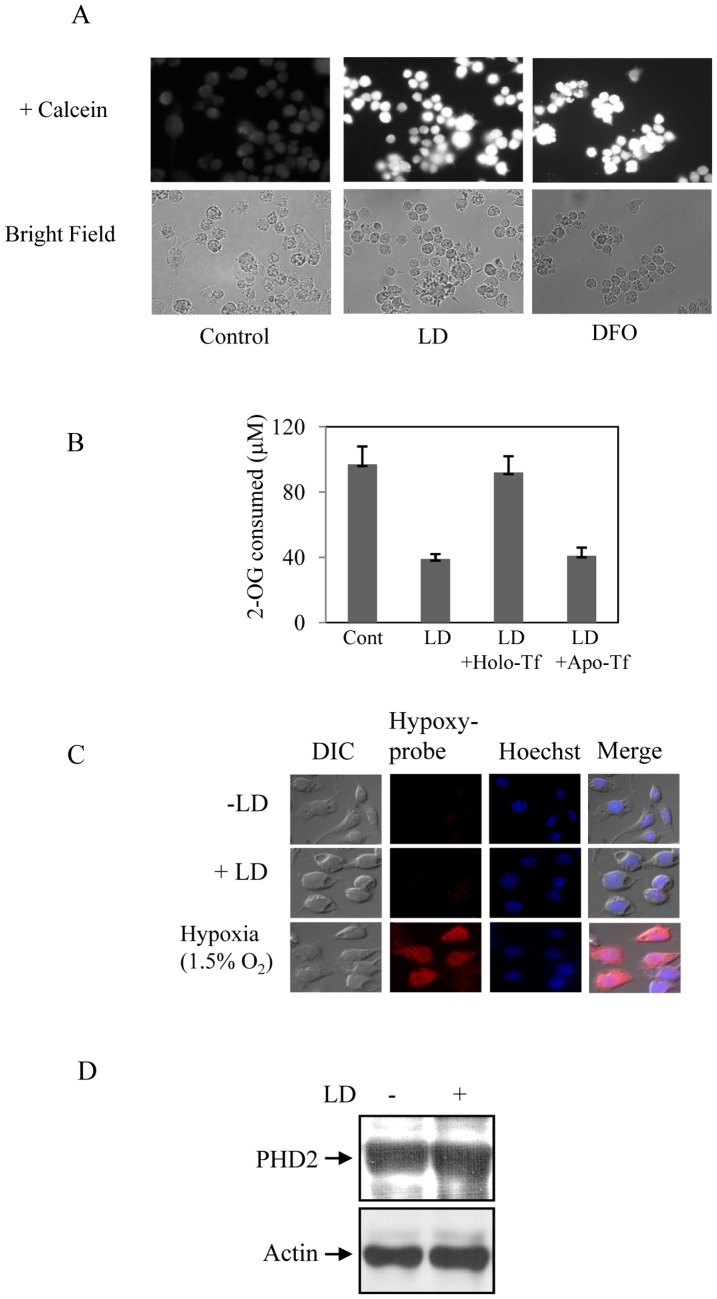
LD depletes cellular iron pool to block PHD activity. A. Labile iron pool was detected in untreated control and LD infected J774 cells (6 h). DFO (100 µM) was used as positive control. B. After 8 h of LD infection apo-Tf (10 µM) and holo-Tf (10 µM) were added for 1 h and then PHD activity was assayed. Result is expressed as standard deviation of four independent experiments. C. J774 cells were infected with LD or exposed to hypoxia (1.5% O_2_) for 6 h and then incubated with 200 µM of pimonidazole hydrochloride (hypoxyprobe-1) for 2 h under similar conditions. Then cells were fixed, washed and immunodetected with mAb to hypoxyprobe for visualization under fluorescence microscope. D. J774 cells were infected with LD for 16 h and PHD2 (upper panel) and actin (lower panel) expressions were determined by Western blot analysis in cell extract. Result is representative of one of the three independent experiments.

Hypoxia, hypoxia mimetic like cobalt chloride, iron chelation and several other inducers promote HIF-1α expression by post-translational stability mechanism [10]. Interestingly, we detected only about 2-fold increase in HIF-1α mRNA in LD-infected J774 cells whereas about 4-fold increase in HIF-1α protein expression was detected in similar condition (Fig. 1B). This led us to hypothesize that LD-infection also might result into HIF-1α stabilization in host cells. To verify this, we tested HIF-1α protein stabilization during LD infection. After 6 h of infection, J774 cells were treated with cycloheximide (5 µg/ml) and rate of HIF-1α degradation was analyzed by Western blot analysis. The half-life of HIF-1α in LD-infected cells was found close to 20 min; whereas, half-life of HIF-1α was detected about 2 min in uninfected cells (Fig. 4A-B) as reported earlier during normoxic condition [1]. Cobalt chloride was used as a positive control to verify HIF-1α stabilization (Fig. 4A–B). These results suggest involvement of HIF-1α stabilization as second mechanism of HIF-1 activation during LD infection. Then, we determined PHD activity in LD infected cells as cellular PHD activity was shown to inversely affect HIF-1α stabilization [4], [5]. Results showed LD infection decreased the activity substantially like the positive control iron chelator deferrioxamine (DFO) (Fig. 4C). Depletion of cellular iron is known to affect prolyl hydroxylase activity *in vitro* and *in vivo* as iron is an important cofactor of HIF-1α hydroxylation by prolyl hydroxylases [25]. Recently, we demonstrated the unique ability of intracellular LD to deplete iron from host labile iron pool (LIP) [26] that could be the cause of decreased prolyl hydroxylase activity. Determination of LIP in LD-infected J774 cells showed a strong depletion of LIP than uninfected cells (Fig. 5A). Iron chelator DFO was used as a positive control (Fig. 5A). To confirm that decrease in PHD activity was actually due to depletion of LIP, LD-infected cells were supplemented with physiological concentration of holo-transferrin or apo-transferrin and cellular PHD activity was determined. Decrease in PHD activity due to LD-infection was reversed by supplementation of only holo-transferrin but not by apo-transferrin (Fig. 5B). This experiment strongly suggests that LD-induced depletion of LIP in host cells could affect the PHD activity as supplementation of iron as holo-transferrin could reverse it. Supplementation of either holo-transferrin or apo-transferrin did not show any significant effect on PHD activity in uninfected cells (data not shown). To verify whether cellular oxygen level had any influence on decrease in LD-induced PHD activity; we exposed cells to hypoxyprobe that is known to sense decrease in cellular oxygen level. No signal with hypoxyprobe was detected in LD-infected J774 cells (Fig. 5C); whereas, exposure of cells to hypoxia (1.5%) showed strong signal with hypoxyprobe suggesting cellular oxygen level was not altered due to LD infection (Fig. 5C). Interestingly, a recent report showed PHD2 expression was affected in host cells due to infection of *Toxoplasma gondii* [27]. When we tested PHD2 expression in LD-infected cells no change was detected even after 16 h of infection (Fig. 5D). All these results strongly suggest that LD activates HIF-1 in host macrophage simultaneously by two distinct mechanisms involving HIF-1α transcription as well as HIF-1α stabilization.

### HIF-1 Activation is Beneficial for Intracellular LD

**Figure 6:**
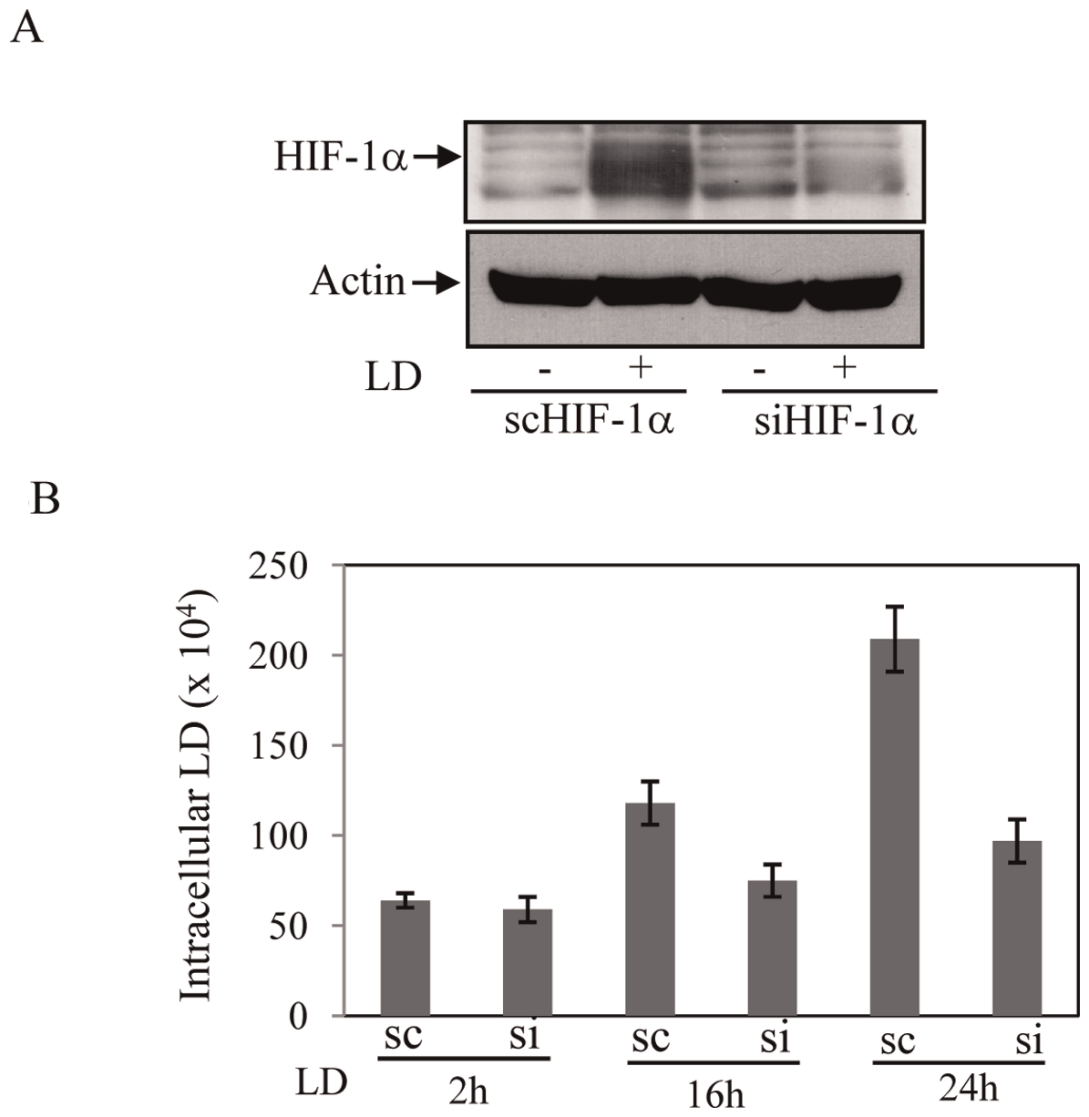
HIF-1 activation is beneficial for intracellular LD. A. Expression of HIF-1α was detected by Western blot analysis in LD infected J774 cells (16 h) those were previously transfected with either HIF-1α specific siRNA (siHIF-1α) or scrambled RNA (scHIF-1α). B. Intracellular LD was isolated and counted at 2 h, 12 h and 24 h of LD infection from siHIF-1α and scHIF-1α transfected J774 cells. Results are representative of three independent experiments performed in duplicates, P<0.06, ANOVA.

To find the role of HIF-1 on the outcome of LD infection; we blocked HIF-1α expression in J774 cells using HIF-1α specific siRNA. The increased expression of HIF-1α was significantly blocked by the specific siRNA (HIF-1-KD); whereas, non-specific siRNA (scRNA) showed no effect on HIF-1α expression (Fig. 6A). The growth of intracellular LD was significantly inhibited in HIF-1-KD J774 cells compared to scRNA transfected cells (Fig. 6B) suggesting beneficial effect of HIF-1 activation on parasite within host macrophage. When number of intracellular LD was counted after 2 h of infection similar numbers of parasite were detected in both the scRNA and siRNA transfected macrophages indicating HIF-1 did not play any role in entry of the parasite but was beneficial for survival in post-infective stage (Fig. 6B).

### HIF-1α Overexpression Promotes Growth of Intracellular LD

**Figure 7:**
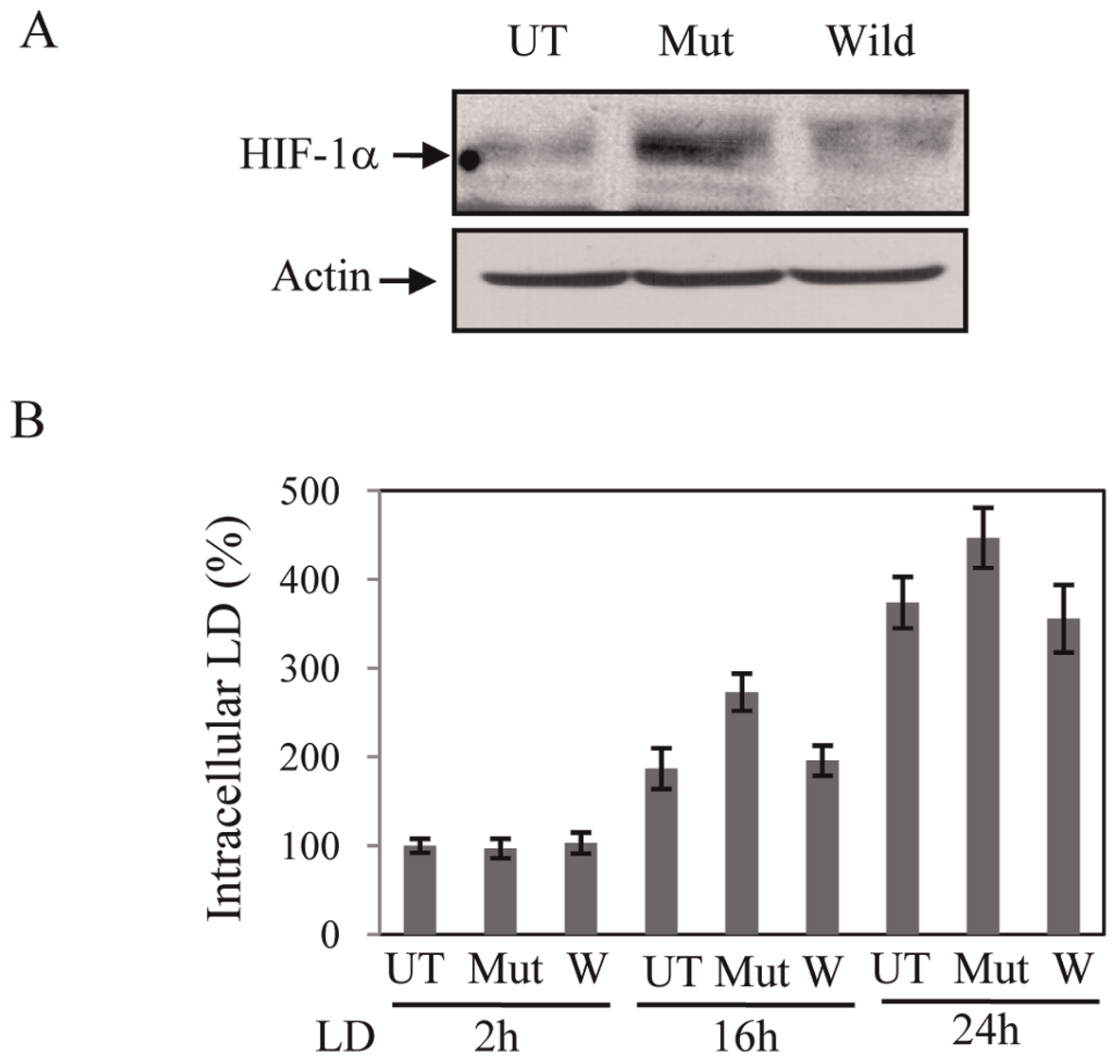
Expression of stable form of HIF-1α promotes intracellular LD growth. J774 cells were transfected with mutated cDNA of HIF-1α (P/A) or wild type HIF-1α cDNA or remained untransfected. Western blot analyses were performed in nuclear extracts for HIF-1α (upper panel) or actin (lower panel). Result is representative of one of the three independent experiments. B. Intracellular LD in J774 cells was counted either after 2 h, 12 h or 24 h of infection in untransfected, mutated HIF-1α (P/A) or wild type HIF-1α cDNA transfected cells. Results are representative of three independent experiments performed in duplicates, P<0.05, ANOVA.

To further verify the role of HIF-1 in LD-infection in to host macrophages; we over-expressed a stable mutant of HIF-1α (HIF-1α P/A) in which pro^402^ and pro^564^ were mutated to alanine (kind gift from Dr. Ritu Kulseshthra). We initially verified that transfection of HIF-1α P/A cDNA actually resulted into increased expression of HIF-1α by Western blot analysis (Fig. 7A, lane 2) than untransfected cells (UT, Fig 7A, lane 1) or transfection of wild type HIF-1α (Wild, Fig 7A, lane 3). Then in a similar condition cells were infected with LD and the number of intracellular LD was counted after 2 h, 12 h and 24 h. We detected a similar number of intracellular LD after 2 h in untransfected, mutant and wild type HIF-1α transfected cells suggesting that HIF-1α amount has no role in infectivity of LD (Fig. 7B) as found in the previous experiment. To further confirm that HIF-1α over-expression did not influence phagocytosis mechanism, we performed phagocytosis assay. In all cases (untransfected, mutant and wild-HIF-1α transfected), a similar number of latex beads was detected within macrophages (data not shown) further supporting that cellular amount of HIF-1α had no influence on the initial infection rate of LD. When intracellular LD was counted after 12 h in HIF-1α over-expressed cells about 70% increase in intracellular LD was detected compared to untransfected or wild-type HIF-1α transfected cells. Higher growth of LD (about 25–30% compared to untransfected or wild-HIF-1α transfected cells) could be observed in HIF-1α over-expressed cells even after 24 h. Reduction in comparable growth rate at 24 h compared to 12 h was probably due to normal HIF-1 activation by LD-infection in untransfected and wild-HIF-1α transfected cells that limited the advantage of HIF-1α over-expression. These experiments further suggest HIF-1α expression is beneficial for intracellular LD.

## Discussion

Mechanism of HIF-1 activation and its role on the outcome of infection of protozoan parasites in macrophage is less understood so far. In this study we demonstrated that unlike most of other infective pathogens, HIF-1 activation in host macrophage is beneficial for survival of the parasite *Leishmania donovani*. Uniquely, this parasite activates HIF-1 by two distinct mechanisms not reported so far for any other pathogens. This implies a crucial role of HIF-1 for the benefit of this intracellular pathogen.

In general, HIF-1 activation during infections caused by *Streptococcus pyogenes*, *Pseudomonas aeruginosa*, *Salmonella typhimurium* and several other bacteria promotes increased killing of these pathogenic bacteria by modulating several innate immune responses by the host [10], [11]. For a few pathogens activation of HIF-1 in host does not lead to their elimination as we detected for LD in this study. *Bartonella henselae* exploits HIF-1 activation in host cells by increasing VEGF expression for inducing angio-proliferative disorders [28]. In general, viral infection is also contained by HIF-1 activation. It was reported that HIF-1 activation by hypoxia or pharmacological agents can suppress the cytolytic injury and viral replication in infections mediated by vesicular stomatitis virus (VSV) by activating TGF-β and other antiviral genes [29]. On the contrary, HIF-1 activation fails to resolve infections caused by hepatitis B and C viruses (HCV and HBV), instead prolonged HIF-1 activation leads to VEGF mediated neovascularization leading into the development of hepatocellular carcinoma [30], [31]. Interestingly, another protozoan parasite *Toxoplasma gondii* activates HIF-1 in host cells by stabilizing HIF-1α by suppressing PHD2 expression for its survival and growth [27] but the precise role of HIF-1 in its survival is not clear yet. Our study suggests that like *T. gondii* LD also exploits activation of HIF-1 in mammalian host for its survival and growth. It will be interesting to find whether all protozoan parasites use HIF-1 for their advantage or only these two parasites are different than others.

HIF-1 is activated mostly by HIF-1α stabilization by various stimuli or by transcription during bacterial infection [10]. HIF-1α transcription is detected as an essential mechanism of HIF-1 activation in response to inflammation and infection [9]–[11]. The basal transcription of HIF-1α is dependent on NFκB [15]. In response to LPS and bacterial infections NFκB binding to HIF-1α promoter is further increased [14], [15]. Amongst infection related HIF-1 activation mechanism enterobacteriaceae infection causes HIF-1α stabilization by secreting iron-chelating siderophore [32]. Respiratory syncytial virus (RSV) also activates HIF-1 in pulmonary epithelia by an oxygen-independent mechanism [33]; however, exact mechanism of HIF-1 activation by RSV is not clear so far. In this study, we observed that LD infection could promote HIF-1α transcription (Fig. 3), although molecular mechanism remained to be understood. NFκB is reported as the main contributor of HIF-1α transcription by LPS treatment or bacterial infection [13], [14]. Moreover, several other physiological stimuli like thrombin and reactive oxygen species generation could activate NFκB for HIF-1α transcription [34]. There are conflicting reports of NFκB activation in host cells during leishmanial infection as increased NFκB activity was reported by both LD infection and by its membrane component lipophosphoglycan (LPG) [35]. In contrary, down regulation of NFκB activity was also reported by LD infection [36]. It will be interesting to find the precise molecular mechanism of HIF-1α transcription during LD infection in macrophages that needs further study.

In this study we revealed that LD infection not only increased HIF-1α transcription but also affected host PHD activity to stabilize HIF-1α (Fig. 4A-C). The cellular PHD activity depends on the availability of cofactors like oxygen or iron [4], [5]. In an earlier report we demonstrated that intracellular LD has a unique ability to deplete labile iron pool (LIP) in host macrophage [26]. This is again confirmed in the current study by using iron-sensitive fluorescence probe calcein-AM (Fig. 5A). It is well known that depletion of cellular iron pool promotes binding of iron sensors IRP1/IRP2 to iron responsive element (IRE). We reported increased IRE-IRP binding in spleen derived macrophages of LD infected mice and J774 macrophages [26] strongly suggesting depletion of host iron pool during LD-infection. To confirm that depletion of iron pool in host by LD was actually the cause of decreased PHD activity, we supplemented physiological concentration of holo-transferrin or apo-transferrin after LD infection and determined PHD activity. PHD activity was reversed only by supplementation of holo-transferrin (Fig. 5B) but not by similar concentration of apo-transferrin. To consider that whether LD infection also could affect cellular oxygen level to decrease PHD activity we performed experiments with hypoxyprobe. Hypoxyprobe is known to react in cellular hypoxic condition as we detected in our experiment with 1.5% oxygen (Fig. 5C). We were not able to detect any hypoxyprobe sensitivity in host macrophage during LD infection suggesting the decrease in PHD activity was only due to iron depletion but not due to depletion of cellular oxygen level. A recent report demonstrated that another protozoan parasite *Toxoplasma gondii* could stabilize HIF-1α by decreasing PHD2 amount in the host cells [27]. Although, three HIF-1α prolyl hydroxylases (PHD1-3) have been identified, gene knockout and siRNA studies suggested PHD2 as primarily responsible for regulating HIF-1α [37]. In our experiment we have not found any decrease in PHD2 protein expression in macrophages by LD infection (Fig. 5D) unlike *T. gondii* suggesting these two protozoan parasites suppress PHD activity by distinctly different mechanisms to exploit benefit of HIF-1 activation in host. Interestingly, the utilization of two distinct mechanisms for HIF-1 activation simultaneously has not been reported for any other infection so far.

We observed that like bacterial infection [10], [11] HIF-1 has no influence on rate of infection of LD as numbers of intracellular LD were similar after 2 h of infection (Fig. 6B) for both HIF-1 containing and KD cells. Similarly, HIF-1α overexpressed cells did not show any significant change in its phagocytic capacity (Fig. 7B). So, the inability of LD growth in HIF-1KD cells depends on its failure to exploit HIF-1-less host environment. Similarly, already present HIF-1 in HIF-1α overexpressed cells provided advantage for intracellular growth of the parasite. The current study does not address the detail cellular mechanism(s) by which LD exploits HIF-1 dependent cellular metabolism of host cells. LD resides and proliferates within individual tight-fitting parasitophorous vacuoles (PV) that contain variety of carbon sources and essential nutrients but poor in hexose accumulation [19]. Previous report of inability of growth and survival of glucose transporter mutants of *L. mexicana* within macrophage [38] suggests that glucose acquisition from host is essential for intracellular LD. Given the role of HIF-1 in glucose metabolism the intracellular alteration and utilization of host glucose metabolism may be advantageous to LD. Our finding of increased GLUT-1 expression in infected macrophages (Fig. 2D) also suggests this hypothesis. We also observed that plasminogen activator inhibitor 1 (PAI-1), a HIF-1 target gene [39] is induced by LD infection in infected macrophages (Fig. 2D). Since, PAI-1 can block apoptosis [40] and one of the survival strategies of leishmania parasite within macrophages is to inhibit apoptosis mechanism of host [41], LD induced PAI-1 by HIF-1 dependent mechanism could be also another potential mechanism for its intracellular survival advantage. There are close to 100 genes are regulated by HIF-1 [42]; we consider LD may exploit several HIF-1 target genes for its survival advantage. It needs a detailed study to understand contributions of specific HIF-1 target genes those are advantageous to intracellular LD.

Interestingly, HIF-1 activation is also responsible for increased synthesis of immune effector molecules like nitric oxide, granule proteases and antimicrobial peptides in phagocytes [11]. Thus, to gain advantage from HIF-1 activation, the parasite should neutralize these immune effector molecules. The unique ability of leishmania to reside within acid rich phagolysosomal vesicles [43] as well as to suppress nitric oxide generation may be crucial for this balancing act [44]. When other invading pathogens are falling prey of HIF-1 mediated immune responses, ability of leishmania to suppress nitric oxide generation may be helpful to exploit HIF-1 mediated alteration of host metabolism. Similarly, when other pathogens are destroyed in lysosomal compartments by acid rich environment and granule proteases, then leishmania could survive by adopting with this extreme environment [43]. Soluble carrier family11, member A1 (SLC11A1, previously known as NRAMP1), a protein-coupled divalent ion transporter was the first infectious disease susceptibility gene identified, whose allelic variation was reported to alter the risk of leishmaniasis [10]. Interestingly, HIF-1 regulates heritable variation and allele expression phenotypes of SLC11A1 from a Z-DNA-forming microsatellite [45]. Given the role of this proton efflux pump in transport of divalent metal ions like Fe^2+^ or Mn^2+^ from lysosomal/phagolysosomal compartment to cytosol, HIF-1 mediated SLC11A1 expression should be detrimental to intracellular LD growth; however, the parasite may overcome this condition by expressing divalent metal ion transporter LIT-1 in its intracellular form [46] during successful infection.

In summary, we found that unlike most of other pathogenic organisms LD utilizes HIF-1 in infected macrophage in its favor for its intracellular survival advantage. This is also the first demonstration of HIF-1 activation by dual mechanism during any intracellular infection. Finding HIF-1 inhibitors is an active area of research because of its central role in angiogenesis and other pathological conditions [47]. Rational design of such an inhibitor thus could also be helpful in controlling this fatal intracellular parasitic infection for which available drugs are mostly resistant.

## Materials and Methods

### Cell Culture

J774 A.1 macrophage cell line (J774) and RAW 264.7 were from ATCC and maintained in RPMI-1640 (Sigma) medium supplemented with 10% heat inactivated fetal bovine serum (Hyclone) and 100 units/ml penicillin, 100 µg/ml streptomycin (Sigma) in a humidified CO_2_ incubator at 37°C as described earlier [26]. Peritoneal macrophages were isolated from BALB/c mice after injecting 2 ml of 2% starch i.p. as described earlier [48]. Splenic macrophages were isolated from BALB/c female mice following the procedure mentioned before [26] and cultured in RPMI-1640 medium supplemented with 10% heat inactivated fetal bovine serum (Hyclone), 100 units/ml penicillin and 100 µg/ml streptomycin.

### Animal

#### Ethics statement

BALB/c female mice 4–12 weeks old were procured (National Centre for Laboratory Animal Sciences, Hyderabad, India) and used for infection and propagation of the virulent strain of *Leishmania donovani* AG83 as approved by the Institutional Animal Ethics Committee-Jawaharlal Nehru University (Institutional Animal Ethics Committee Code No. 10/2004) by passaging every 4 weeks.

After infection for 4 weeks spleens of BALB/c mice were removed aseptically, homogenized under sterile conditions and suspended in M199 with 30% FBS. This suspension was incubated at 22°C for 48 to 72 hours. Freshly transformed promastigotes were verified under the microscope and counted; the suspension was centrifuged at 1000 rpm for 10 min at 4°C to remove splenic debris and the promastigotes were centrifuged down at 5000 rpm for 15 min at 4°C. The pellet was resuspended in PBS (pH 7.4) at a concentration of 10^8 ^cells/ml. 100 µl of this freshly transformed promastigotes was again injected in the tail vein of 2–4 weeks old mice.

### Parasite Culture and Maintenance

*Leishmania donovani* parasites in the promastigote stage were maintained in M199 medium supplemented with 10% FBS (Hyclone), 100 units/ml penicillin and 100 µg/ml streptomycin (Sigma) at 22°C in a BOD incubator. To subculture a small aliquot of the stationary phase parasite was added to fresh medium.

### Western Blot Analysis

Nuclear extract was prepared from J774 cells as described earlier [24]. Briefly, 1 × 10^8^ cells were washed with ice-cold phosphate-buffered saline and then with a solution containing 10 mM Tris-HCl, pH 7.8, 1.5 mM MgCl_2_, and 10 mM KCl, supplemented with a protease inhibitor mixture containing 0.5 mM dithiothreitol, 0.4 mM phenylmethylsulfonyl fluoride, and 2 µg/ml each of leupeptin, pepstatin and aprotinin. After incubation on ice for 10 min cells were lysed by 10 strokes with a Dounce homogenizer and the nuclei were pelleted. The pellet was resuspended in a solution containing 420 mM KCl, 20 mM Tris-HCl, pH 7.8, 1.5 mM MgCl_2_, and 20% glycerol, supplemented with the protease mixture described above, and incubated at 4°C with gentle agitation. The nuclear extract was centrifuged at 10,000×g for 10 min, and the supernatant was dialyzed twice against a solution of 20 mM Tris-HCl, pH 7.8, 100 mM KCl, 0.2 mM EDTA, and 20% glycerol. Protein concentration was determined using the Bio-Rad reagent with bovine serum albumin as standard. Proteins (30 µg) were subjected to electrophoresis and transferred to PVDF membrane, incubated with anti HIF- 1α IgG (1∶2000; Novus Biologicals or Abcam), HIF-1β IgG (1∶2000; Novus Biologicals), PHD2 (1∶2000; Abcam), actin (1∶1000) (SantaCruz) or lamin (1∶1000) (SantaCruz) followed by peroxidase conjugated secondary antibody (1∶5000). The specific band was detected by chemiluminescence using ECL reagent.

### Northern Blot Analysis

Total RNA was isolated using TriPure reagent (Roche) and 20 µg RNA was denatured in formamide/formaldehyde, electrophoresed through 1% agarose gel containing 6% formaldehyde and blotted onto nylon membrane. After cross-linking, filters were hybridized to HIF-1α cDNA (Novus Biologicals) labeled by random priming with [α-^32^P] dCTP using a New England Biolab Kit.

### Reverse Transcription Polymerase Chain Reaction Analysis (RT)-PCR

Real-time RT-PCR (Applied Biosystem; 7500 Real Time PCR System) was used to analyze transcripts levels of HIF-1α, VEGF, PAI-1 and GLUT-1 in LD infected cells. Total RNA was insolated using Tripure (Roche, Germany) to perform real-time RT-PCR. cDNA was prepared using 5 µg of total RNA using High capacity cDNA Reverse Transcription kit (Applied Biosystems, USA). Real time RT-PCR for HIF-1α was performed using HIF-1α assay mix (Mm01283760_m1 HIF-1α) procured from Applied Biosystem, and results were normalized using actin as an endogenous control [Mouse ACTB(20X) pre developed TaqMan® Assay Reagents]. Program for HIF-1α amplification was 50°C- 2min; 95°C- 10 min; increasing cycles of (95°C - 15 sec; 60°C- 1 min). For HIF-1α regulated genes VEGF, PAI-1 and GLUT-1 real time RT-PCR was performed using Power SyBr Green PCR master Mix (Applied Biosystem). The PCR reaction contained 30pM each of Forward and Reverse primers of VEGF, PAI-1 and GLUT-1. The following primers were used VEGF: For 5′TAC TGC TGT ACC TCC ACC ATG 3′, Rev 5′ CTT TCT CCG CTC TGA ACA AG 3′; PAI-1: For 5′ AAA GGC ATA CCA AAG GTA TG 3′, Rev 5′ ACT TCA GTC TCC AGA GAG AAC 3′; GLUT-1: For 5′ GGA TCC ATG ATG AAC CTG TTG 3′, Rev 5′ CTC GAG GTG CAG GGT CCG TC 3. Results were normalized using β-actin as endogenous control using following primers- Forward 5′ GAC TGG GAG AAG ATC 3′ and Reverse 5′ GAA TGT AGT TTC ATG 3′. The condition of amplification for VEGF, PAI-1 and Glut-1 was 50°C - 2 min; 95°C-10 min; increasing cycles of (95°C -15 sec, 54°C - 30 sec, 60°C-1 min).

### HIF-1α Promoter and Other Construct Preparation

HIF-1α promoter region (–1034 to +339 of transcription start site) was cloned by PCR from mouse genomic DNA using primers containing Kpn1site in forward primer (5′ ATA CAT GGT ACC CAC GAA GTG TTC CTT TG 3′) and Xho1site in reverse primer (5′ ATA CAT CTC GAG AAA GAG ACA AGT CCA 3′). PCR fragment was cloned into upstream of pGL3 basic vector and confirmed by sequencing. The cloning of functional hypoxia responsive element of ceruloplasmin (CpHRE) and mutated CpHRE (mutHRE) was described before [22].

### Transfection and Reporter Gene Assay

In J774 cells transfection was performed (either CpHRE, mutCpHRE or HIF-1α promoter construct) using Fugene 6 (Roche) according to company’s protocol. Transfected cells were infected with LD. Luciferase activity in cell lysate was assayed using a kit (Promega). As a control of transfection efficiency, cells were also transfected with CMV promoter containing β-galactosidase construct and assay was performed using a kit (Promega). Results are expressed after normalization with β-galactosidase activity.

### Fluorescence Microscopy

For immunofluorescence; LD infected J774, RAW264.7, peritoneal macrophage or macrophages isolated from spleen of age matched control and LD infected mice were fixed, after blocking cells were incubated with primary antibody for 1 h at room temperature. After washing secondary antibody coupled to Cy 3 conjugate (1∶1000) was used for 1 h at room temperature. Antibody was used at following dilution: HIF-1α (1∶1000). After appropriate washing and mounting, cells were visualized under a Zeiss Imager Z1 apotome microscope. Images were captured using a cooled monochrome CCD camera AxioCam HRM using Axiovision Rel 4.8.1 software.

### Assessment of Calcein-sensitive Labile Iron Pool

Labile iron pool was assessed using Calcein-AM as described earlier [26]. In short, LD infected J774 cells were washed with ice-cold PBS and kept in RPMI-1640 (without phenol red). After adding calcein-AM (0.5 µM) cells were incubated at 37°C for 20 minutes. Fluorescence microscopy was done at 488 nm excitation and 517 nm emission. Nikon upright fluorescence microscope model 80i equipped with water emersion objectives and connected with cooled CCD digital camera was used for imaging.

### Detection of Intracellular Hypoxic Condition by using Hypoxyprobe

J774 cells were infected with LD (MOI- 1∶10) for 8 h. Infected cells were incubated with 200 µM of pimonidazole hydrochloride (hypoxyprobe-1, Chemicon) for 2h. Cells were fixed with 4% para-formaldehyde for 10 min and washed twice with 1x PBS. Non specific binding was blocked by incubating cells with 1% BSA solution prepared in 1x PBS. Cells were immunostained with mouse monoclonal antibody (1∶40) against pimonidazole adducts for 1h at room temperature, followed by three washes with 1x PBS. Then cells were further incubated with 1∶1000 dilution of Cy3 tagged anti-mouse secondary antibody followed by three washes with 1x PBS. Cells were mounted over 10% glycerol solution in 1xPBS and visualized under a Nikon Eclipse 80i fluorescence microscope.

### HIF-1α Silencing by siRNA

Silencing of HIF-1α in J774 cells was carried out by transfection of cells with HIF-1α siRNA (from Qiagen, targeted sequence AAC ACA CAG CGG AGC TTT TTT). Same sequence in scramble (CAG AAC CGG ACA TTT AGC TTT) was used for control transfection (HIF-1α scRNA). Two other HIF-1α specific siRNAs from Santa Cruz Biotechnology (sc 44308 and sc 35562) were also tested to silence HIF-1α expression. HIF-1α siRNA obtained from Qiagen and sc44308 could block HIF-1α expression more than 80% and hence used for all related experiments. All the transfections were carried out using company specific protocol and reagents only. For HIF-1α siRNA (sc44308) transfection, control scramble siRNA was also obtained (sc-37007) from Santa Cruz Biotechnology.

### Prolyl Hydroxylase Assay

Prolyl hydroxylase activity was determined by monitoring depletion of 2-oxoglutarate by its post-incubation derivatization with o-phenylenediamine to form a product amenable to fluorescence analysis [34], [49]. The assay was carried out by mixing 1 mM DTT, 0.6 mg/ml catalase, 2-oxoglutarate (2-OG, 500 µM), 200 µM peptide (19 mer of HIF-1α, 556–574, DLDLEMLAPYIPMDDDFQL) and 50 mM Hepes pH 7.5 at 37°C for 5 min. The reaction was initiated by addition of cytosolic extract (50 µg)/iron mix to the substrate/cofactor mix in a final volume of 100 µl. After 5 min, 200 µl of 0.5 M HCl was added to stop the reaction. Derivatization was achieved by addition of 100 µl of 10 mg/ml OPD in 0.5 M HCl for 10 min at 95°C. After 5 min centrifugation, supernatant (50 µl) was made basic by adding 30 µl of 1.25M NaOH and then fluorescence was measured using excitation at 340 nm and emission at 420 nm.

### Counting of Intracellular Parasites

The intracellular parasites from J774 cells were counted using percoll gradient as described earlier [26], [50]. Briefly, infected macrophages were lysed by 4 freeze-thaw cycles. Then cell lysates were put in an individual percoll gradient (in the order of 90%, 40% and 20%) and spun at 800 xg for 1 hour. The band at the interface of 90/40% percoll is collected and the volume of each collection is equilibrated up to 1 ml. Parasites were counted in the improved Neubaur Counting Chamber.
